# Decreased Platelet Inhibition by Thienopyridines in Hyperuricemia

**DOI:** 10.1007/s10557-020-07058-x

**Published:** 2020-08-26

**Authors:** Silvia Lee, Patricia P. Wadowski, Timothy Hoberstorfer, Constantin Weikert, Joseph Pultar, Christoph W. Kopp, Simon Panzer, Thomas Gremmel

**Affiliations:** 1 Department of Internal Medicine II, Medical University of Vienna, Vienna, Austria; 2 Department of Blood Group Serology and Transfusion Medicine, Medical University of Vienna, Vienna, Austria; 3 Department of Internal Medicine I, Landesklinikum Mistelbach-Gänserndorf, Mistelbach, Austria

**Keywords:** Hyperuricemia, Clopidogrel, Prasugrel, Ticagrelor, Platelet reactivity

## Abstract

**Purpose:**

Hyperuricemia carries an increased risk of atherothrombotic events in acute coronary syndrome (ACS) patients undergoing percutaneous coronary intervention (PCI). This may at least in part be due to inadequate P2Y12 inhibition. The aim of this study was to prospectively investigate the potential association between hyperuricemia and decreased platelet inhibition by P2Y12 antagonists.

**Methods:**

Levels of uric acid as well as on-treatment residual platelet reactivity in response to adenosine diphosphate (ADP) were assessed in 301 clopidogrel-treated patients undergoing elective angioplasty and stenting, and in 206 prasugrel- (*n* = 118) or ticagrelor-treated (*n* = 88) ACS patients following acute PCI. Cut-off values for high on-treatment residual ADP-inducible platelet reactivity (HRPR) were based on previous studies showing an association of test results with clinical outcomes.

**Results:**

Hyperuricemia was significantly associated with increased on-treatment residual ADP-inducible platelet reactivity in clopidogrel- and prasugrel-treated patients in univariate analyses and after adjustment for differences in patient characteristics by multivariate regression analyses. In contrast, ticagrelor-treated patients without and with hyperuricemia showed similar levels of on-treatment residual platelet reactivity to ADP. HRPR occurred more frequently in clopidogrel- and prasugrel-treated patients with hyperuricemia than in those with normal uric acid levels. In contrast, hyperuricemic patients receiving ticagrelor did not have a higher risk of HRPR compared with those with normal uric acid levels.

**Conclusion:**

Hyperuricemia is associated with decreased platelet inhibition by thienopyridines but a normal response to ticagrelor. It remains to be established if lowering uric acid increases the antiplatelet effects of clopidogrel and prasugrel in hyperuricemic patients with HRPR.

## Introduction

Uric acid is generated through oxidation of hypoxanthine to xanthine and further degradation of xanthine by xanthine oxidase and constitutes the final product of purine catabolism. Levels of uric acid are associated with chronic kidney disease, diabetes, arterial hypertension, and metabolic syndrome [1–3]. Moreover, by enhancing intracellular oxidative stress, endothelial dysfunction, and vascular inflammation[4–7], high levels of uric acid may contribute to the development and progression of cardiovascular disease [1]. Indeed, hyperuricemia is increasingly recognized as risk factor for adverse ischemic events in atherosclerosis. Recently, Tscharre et al. found elevated uric acid to predict myocardial infarction and cardiovascular death in 1215 ACS patients undergoing percutaneous coronary intervention (PCI) over a follow-up period of 5.5 years [8]. Furthermore, Wong et al. reported that high serum uric acid concentrations were linked to an increased risk of cardiac death in 354 stroke survivors [9]. These observations point towards a prothrombotic milieu in hyperuricemia. We therefore hypothesized that hyperuricemia is associated with enhanced on-treatment residual platelet reactivity in cardiovascular disease. To investigate whether or not current state-of-the-art P2Y12 receptor inhibition is able to adequately suppress platelet aggregation in hyperuricemic patients, we studied on-treatment residual adenosine diphosphate (ADP)-inducible platelet reactivity by various platelet function tests in patients without and with hyperuricemia following elective and acute PCI.

## Methods

### Patients

In this prospective cohort study, 301 patients undergoing elective angioplasty and stenting (group 1) and 206 ACS patients undergoing acute PCI (group 2) were enrolled at the Department of Internal Medicine II of the Medical University of Vienna. All patients of group 1 were on chronic aspirin therapy and had received 100 mg aspirin per day for at least 2 weeks prior to study enrolment. Except 69 patients (22.9%) on clopidogrel maintenance therapy, all patients received a loading dose of 300 mg clopidogrel 24 h prior to intervention (*n* = 152; 50.5%) or a loading dose of 600 mg clopidogrel on the day of intervention at least 2 h prior to angioplasty (*n* = 80; 26.6%) followed by a daily dose of 75 mg clopidogrel. Patients of group 2 received 100 mg aspirin per day plus 10 mg prasugrel once daily (118 patients) or 90 mg ticagrelor twice daily (88 patients).

Exclusion criteria were a known aspirin, clopidogrel, prasugrel, or ticagrelor intolerance (allergic reactions, gastrointestinal bleeding); a therapy with vitamin K antagonists (warfarin, phenprocoumon, acenocoumarol) or direct oral anticoagulants (dabigatran, rivaroxaban, apixaban and edoxaban); a treatment with ticlopidine, dipyridamol, or nonsteroidal antiinflammatory drugs; a family or personal history of bleeding disorders, malignant paraproteinemias, myeloproliferative disorders, or heparin-induced thrombocytopenia; severe hepatic failure; known qualitative defects in thrombocyte function; a major surgical procedure within 1 week before enrollment; a platelet count < 100.000 or > 450.000/μl; and a haematocrit < 30%.

The study protocol was approved by the Ethics Committee of the Medical University of Vienna in accordance with the Declaration of Helsinki, and written informed consent was obtained from all study participants.

### Blood Sampling

Blood was drawn by aseptic venipuncture from an antecubital vein using a 21-gauge butterfly needle (0.8 × 19 mm; Greiner Bio-One, Kremsmünster, Austria) 1 day after elective intervention in group 1 and 3 days after acute PCI in group 2, as previously described [10].

### Measurement of Uric Acid

Serum levels of uric acid were measured in the central laboratory of the Medical University of Vienna according to standardized protocols. Hyperuricemia was defined as uric acid > 6 mg/dL in women and > 7 mg/dL in men, respectively.

### Determination of Glycoprotein IIb/IIIa Activation

The binding of the monoclonal antibody PAC-1 to activated GPIIb/IIIa was determined in citrate-anticoagulated blood after exposure to 1 μM ADP (Roche Diagnostics GmbH, Mannheim, Germany), as previously published [11]. At acquisition, the platelet population was identified by its characteristics in the forward scatter versus side scatter plot. A total of 10,000 events were acquired within this gate. This population was further identified by platelets stained with the platelet-specific monoclonal antibody anti-CD42b versus side scatter. Binding of the antibody against activated GPIIb/IIIa was determined in a histogram for PAC-1. The mean fluorescence intensity (MFI) based on all events was used for statistical calculations. Platelet surface expression of activated GPIIb/IIIa in response to ADP was available for 288 patients (95.7%) of group 1.

### Measurement of Soluble CD40 Ligand

Soluble CD40 ligand (sCD40L) was measured in duplicates with a commercially available ELISA (Quantikine®, R&D Systems, Minneapolis, MN), following the manufacturer’s instructions. sCD40L was available for 204 patients of group 1 (67.8%).

### Light Transmission Aggregometry

Light transmission aggregometry (LTA) was performed on a PAP-8E aggregometer (Bio-Data, Horsham, PA USA) as previously described [10]. Platelet counts were not adjusted as the median platelet counts were 208 G/L (176–250 G/L) and 222 G/L (192–258 G/L) in groups 1 and 2, respectively. The optical density of platelet poor plasma was set as 100% aggregation. Platelet aggregation was initiated by addition of 10 μM ADP to platelet rich plasma. Optical density changes were recorded photoelectrically for 10 min as platelets began to aggregate to obtain maximal aggregation %. LTA in response to ADP was available for 299 patients (99.3%) of group 1 and for all patients (100%) of group 2.

### VerifyNow P2Y12 Assay

The VerifyNow P2Y12 assay was performed as previously described [10]. With this assay, higher P2Y12 Reaction Units (PRU) reflect greater ADP-inducible platelet reactivity. The VerifyNow P2Y12 assay was available for 300 patients (99.7%) of group 1.

### Multiple Electrode Aggregometry

Impedance aggregometry was performed with the Multiplate analyzer (Roche Diagnostics, Mannheim, Germany) as previously described [10]. After addition of 6.4 μM ADP (Roche Diagnostics, Mannheim, Germany) to hirudin-anticoagulated blood, the adhesion of activated platelets to the electrodes led to an increase in impedance, which was detected for each sensor unit separately and transformed to aggregation units (AU) that were plotted against time. One AU corresponds to 10 AU ∗ min (area under the curve of AU). Multiple electrode aggregometry (MEA) in response to ADP was available for 293 patients (97.3%) of group 1 and for all patients (100%) of group 2.

### Vasodilator-Stimulated Phosphoprotein Phosphorylation Assay

For determination of the platelet reactivity index (PRI), the extent of vasodilator-stimulated phosphoprotein (VASP) phosphorylation was measured by MFI values in the presence of PGE1 without (T1) or with ADP (T2). After subtraction of the background fluorescence from the corresponding fluorescence values, the PRI (%) was calculated according to the following formula:

$$ \mathrm{PRI}\%=\left[\left[\mathrm{T}1\left(\mathrm{PGE}1\right)-\mathrm{T}2\left(\mathrm{PGE}1+\mathrm{ADP}\right)\right]/\mathrm{T}1\left(\mathrm{PGE}1\right)\right]\times 100 $$

The VASP assay was available for 299 patients (99.3%) of group 1.

### Genotyping

Genomic DNA for genetic analysis was isolated from 400 μl EDTA blood with the MagNA Pure DNA-isolation system (Roche Diagnostics) according to the manufacturer’s instructions. Cytochrome (CYP) 2C9 genotyping was performed using the LightMix for the detection of human CYP2C9*2 and CYP2C9*3 variants (TIB MolBiol), LightCycler FastStart DNA Master Hybridisation Probes (Roche), and the LightCycler 2.0 capillary PCR machine, as previously described [12]. Allelic variants of CYP2C19 were determined with the Infiniti® CYP450 2C19+ assay (AutoGenomics, Carlsbad, CA, USA), as previously described [13]. Genotyping of CYP2C9 and CYP2C19 loss-of-function polymorphisms was available for 278 patients (92.4%) of group 1.

### Sample Size Calculation and Statistical Analysis

A sample size calculation was based on the observed mean ± SD of ADP-inducible platelet reactivity by the VerifyNow P2Y12 assay (204 ± 89 PRU) in a former population of 80 patients on dual antiplatelet therapy with aspirin and clopidogrel 24 h after elective angioplasty and stenting [10]. We calculated that we needed to include 300 patients to be able to detect a 20% relative difference of ADP-inducible platelet reactivity by the VerifyNow P2Y12 assay between patients without and with hyperuricemia with a power of 96% (using a two-sided alpha level of 0.05).

Statistical analysis was performed using the Statistical Package for Social Sciences (IBM SPSS version 26, Armonk, New York, USA). Median and interquartile range of continuous variables are shown. Categorical variables are given as number (%). We performed Mann–Whitney *U* tests to detect differences of continuous variables. The chi-square test and the Fisher´s exact test were used to detect differences in categorical variables, respectively. Multivariate linear regression analyses were used to adjust for patient characteristics that were different between patients without and with hyperuricemia. Covariates for adjustment were selected on the basis of univariate analyses (*p* ≤ 0.1), including age, sex, body mass index (BMI), hypertension, hyperlipidemia, diabetes, active smoking, hemoglobin, white blood cell count (WBC), platelet count, serum creatinine, high-sensitivity C-reactive protein, use of aspirin, allopurinol, statins, angiotensin converting enzyme inhibitors or angiotensin receptor blockers, beta-blockers, proton pump inhibitors, and calcium channel blockers. Two-sided *p*-values < 0.05 were considered statistically significant.

## Results

Hyperuricemia was seen in 83 (27.6%) and 42 (20.4%) patients in groups 1 and 2, respectively. Characteristics of patients without and with hyperuricemia in groups 1 and 2 are shown in Tables 1 and 2.

**Table 1:** Clinical and laboratory characteristics of clopidogrel-treated patients (*n* = 301) without and with hyperuricemia

Characteristics	No hyperuricemia (*n* = 218)	Hyperuricemia (*n* = 83)	*p*
Demographics			
Age, years	64 (57–73)	67 (61–78)	0.005
Male sex	146 (67)	53 (63.9)	0.61
BMI (kg/m^2^)	26.2 (24.1–29.1)	28.1 (25–31.1)	0.002
Medical history			
Hypertension	190 (87.2)	82 (98.8)	0.002
Hyperlipidemia	204 (93.6)	75 (90.4)	0.34
Diabetes mellitus	68 (31.2)	28 (33.7)	0.67
Active smoking	98 (45)	31 (37.3)	0.23
Laboratory data			
Hemoglobin (g/dL)	13 (11.5–14.3)	12.9 (11.4–14.2)	0.3
WBC (G/L)	8.5 (6.9–10.5)	8.1 (6.7–10)	0.32
Platelet count (G/L)	209 (175–250)	208 (176–250)	0.86
Serum creatinine (mg/dL)	1 (0.9–1.1)	1.2 (1–1.5)	< 0.001
hsCRP (mg/dL)	0.8 (0.3–1.6)	1 (0.3–2.5)	0.2
Medication			
Aspirin	218 (100)	83 (100)	1
Allopurinol	15 (6.9)	6 (7.2)	0.92
Statin	211 (96.8)	75 (90.4)	0.03
ACE inhibitor/ARB	186 (85.3)	75 (90.4)	0.25
Beta blocker	147 (67.4)	62 (74.7)	0.22
PPI	113 (51.8)	44 (53)	0.86
CCB	60 (27.5)	34 (41)	0.03

Continuous data are shown as median (interquartile range). Dichotomous data are shown as *n* (%).

*ACE* angiotensin converting enzyme, *ARB* angiotensin receptor blocker, *BMI* body mass index, *CCB* calcium channel blocker, *hsCRP* high-sensitivity C-reactive protein, *PPI* proton pump inhibitor, *WBC* white blood cell count

**Table 2:** Clinical, laboratory, and procedural characteristics of prasugrel (*n* = 118)- and ticagrelor (*n* = 88)-treated patients without and with hyperuricemia

Characteristics	Prasugrel			Ticagrelor		
	No hyperuricemia (*n* = 98)	Hyperuricemia (*n* = 20)	*p*	No hyperuricemia (*n* = 66)	Hyperuricemia (*n* = 22)	*p*
Demographics						
Age, years	56.1 (50.9–61.7)	57 (37.8–70.6)	0.82	56.8 (50–68.8)	64.9 (58.8–73)	0.05
Male sex	78 (79.6)	19 (95)	0.12	56 (84.8)	12 (54.5)	0.003
BMI (kg/m^2^)	27.8 (25.2–30.4)	28.1 (24.1–32)	0.65	26.8 (24.2–29.7)	27 (25.7–30.7)	0.43
Medical history						
Hypertension	62 (63.3)	15 (75)	0.31	46 (69.7)	16 (72.7)	0.79
Hyperlipidemia	76 (77.6)	16 (80)	0.81	48 (72.2)	18 (81.8)	0.39
Diabetes mellitus	17 (17.4)	7 (35)	0.12	21 (31.8)	6 (27.3)	0.69
Active smoking	56 (57.1)	13 (65)	0.52	38 (57.6)	9 (40.9)	0.18
Laboratory data						
Hemoglobin (g/dL)	13.9 (13.1–14.7)	14 (13.3–15.4)	0.46	14 (12.7–14.6)	13.3 (11.9–14.6)	0.4
WBC (G/L)	8.9 (7.9–10.2)	9.8 (7.8–11.8)	0.29	9.2 (6.6–10.5)	9.5 (7.3–10.6)	0.59
Platelet count (G/L)	224 (197–253)	194 (172–219)	0.01	223 (186–265)	238 (215–269)	0.15
Serum creatinine (mg/dL)	0.9 (0.8–1)	1.1 (0.9–1.2)	<0.001	1 (0.8 − 1.1)	1.2 (0.8–1.5)	0.02
hsCRP (mg/dL)	1.2 (0.7–3.6)	2 (0.5–6)	0.63	1.1 (0.4–3.7)	1.5 (1–3.5)	0.36
Medication						
Aspirin	98 (100)	20 (100)	1	66 (100)	22 (100)	1
Allopurinol	2 (2)	2 (10)	0.13	2 (3)	1 (4.5)	1
Statin	97 (99)	20 (100)	0.65	65 (98.5)	22 (100)	1
ACE inhibitor/ARB	94 (95.9)	20 (100)	1	64 (97)	20 (90.9)	0.26
Beta blocker	96 (98)	18 (90)	0.13	65 (98.5)	21 (95.5)	0.44
PPI	75 (76.5)	14 (70)	0.57	43 (65.2)	17 (77.3)	0.29
CCB	6 (6.1)	4 (20)	0.07	5 (7.6)	5 (22.7)	0.11

Continuous data are shown as median (interquartile range). Dichotomous data are shown as *n* (%).

*ACE* angiotensin converting enzyme, *ARB* angiotensin receptor blocker, *BMI* body mass index, *CCB* calcium channel blocker, *hsCRP* high-sensitivity C-reactive protein, *PPI* proton pump inhibitor, *WBC* white blood cell count

In group 1, patients with hyperuricemia were significantly older, had higher serum creatinine levels and a greater BMI than patients without hyperuricemia. Moreover, hypertension and the use of calcium channel blockers were more frequent in patients with hyperuricemia, whereas statins were prescribed more often in patients without hyperuricemia (Table 1; all *p* < 0.05). CYP2C9 and CYP2C19 loss-of-function polymorphisms were present to a similar extent in patients without and with hyperuricemia (CYP2C9: 33 (16.4%) vs. 8 (10.4%) patients; CYP2C19: 58 (28.9%) vs. 28 (36.4%) patients; both *p* > 0.2).

In group 2, there was a trend towards higher levels of uric acid in patients on ticagrelor compared with those on prasugrel (5.8 mg/dL (5.1–7 mg/dL) vs. 5.6 mg/dL (4.5–6.5 mg/dL), *p* = 0.05). Moreover, hyperuricemia was seen in 22 out of 88 ticagrelor-treated patients (25%), but only in 20 out of 118 prasugrel-treated patients (16.9%; *p* = 0.16). Prasugrel-treated patients with hyperuricemia had higher serum creatinine levels and lower platelet counts than those without hyperuricemia (Table 2; both *p* < 0.05). Ticagrelor-treated patients with hyperuricemia had higher serum creatinine levels and were more often women than those without hyperuricemia (Table 2; both *p* < 0.05).

**Fig. 1:**
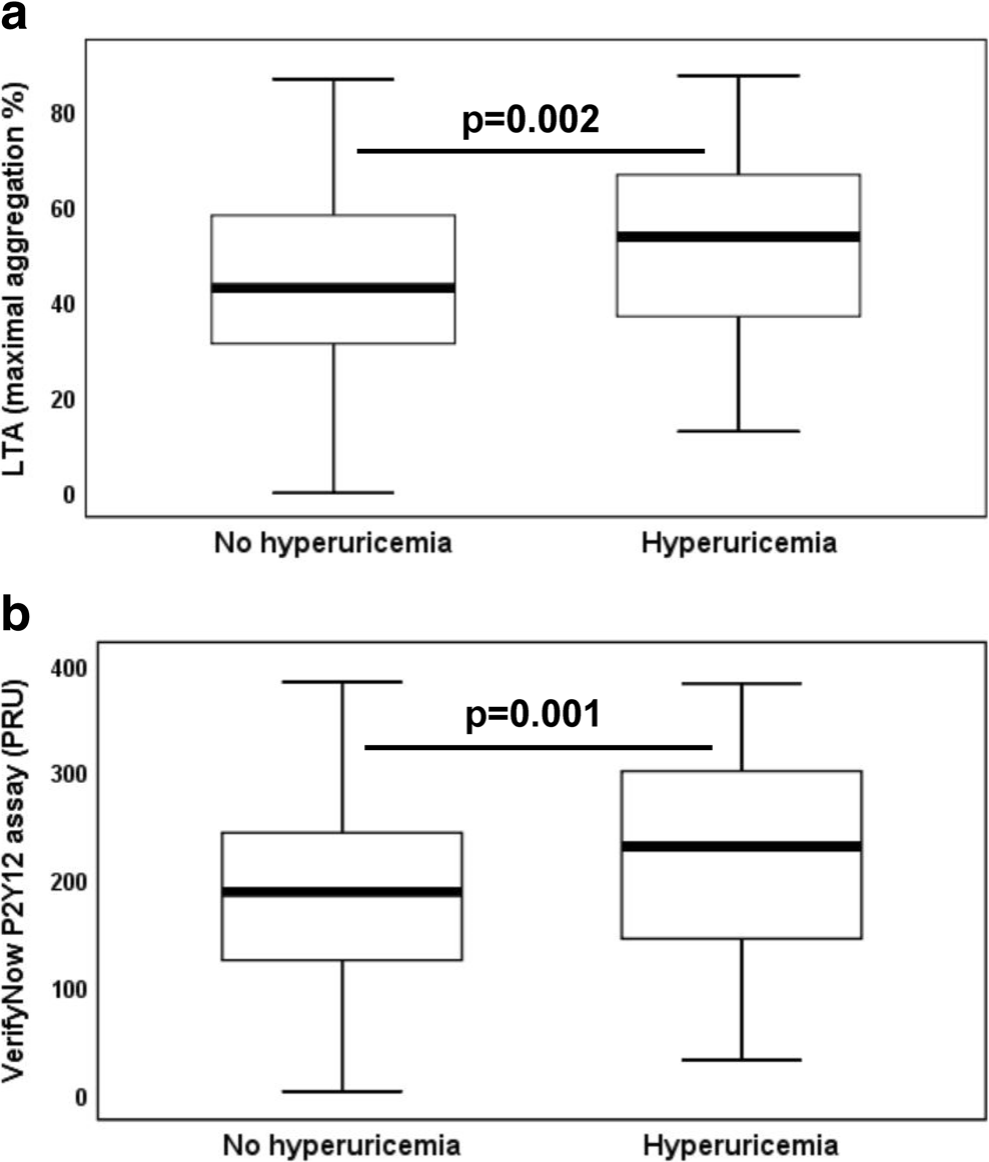
On-treatment residual adenosine diphosphate-inducible platelet reactivity by **a** light transmission aggregometry and **b** the VerifyNow P2Y12 assay in clopidogrel-treated patients without and with hyperuricemia. The boundaries of the box show the lower and upper quartile of data, and the line inside the box represents the median. Whiskers are drawn from the edge of the box to the highest and lowest values that are outside the box but within 1.5 times the box length

In group 1, patients with hyperuricemia showed a significantly higher platelet surface expression of activated GPIIb/IIIa in response to ADP and higher levels of sCD40L than those without hyperuricemia (activated GPIIb/IIIa: 13.7 MFI (8–22 MFI] vs. 9.7 MFI (6.2–14.6 MFI), *p* < 0.001; sCD40L: 277.1 pg/mL (181.4–493.3 pg/mL) vs. 171.7 pg/mL (95.1–320.8 pg/mL), *p* = 0.002). Furthermore, on-treatment residual ADP-inducible platelet reactivity by LTA, the VerifyNow P2Y12 assay, and the VASP assay was significantly higher in patients with hyperuricemia (LTA: Fig. 1a; 53.8% (37–66.7%) vs. 42.9% (31.3–58.2%), *p* = 0.002; VerifyNow P2Y12 assay: Fig. 1b; 232 PRU (143–303PRU) vs. 190 PRU (126–245 PRU), *p* = 0.001; VASP assay: 53.4% (33.9–67.5%) vs. 43% (25.1–58.9%), *p* = 0.002), and there was a trend towards higher platelet aggregation by MEA in response to ADP as compared with patients without hyperuricemia (45 AU (32–60 AU) vs. 38 AU (27–55 AU), *p* = 0.06).

After adjustment for differences in patient characteristics by multivariate regression analyses, hyperuricemia was significantly associated with platelet surface expression of activated GPIIb/IIIa, levels of sCD40L, and on-treatment residual ADP-inducible platelet reactivity by LTA, MEA, and the VASP assay (Table 3). In a second step, we stratified the patient population of group 1 in patients without and with high on-treatment residual ADP-inducible platelet reactivity (HRPR) by the different platelet function tests according to the consensus documents on the definition of high on-treatment platelet reactivity to ADP [14, 15]. The respective cut-off values for HRPR were a maximal aggregation ≥ 70% for LTA, PRU > 208 for the VerifyNow P2Y12 assay, AU > 46 for MEA, and a PRI > 50% for the VASP assay. With use of these thresholds, HRPR was seen in 34 (11.4%), 135 (45%), 109 (37.2%), and 137 (45.8%) patients by LTA, the VerifyNow P2Y12 assay, MEA, and the VASP assay, respectively. HRPR by all assays occurred more frequently in patients with hyperuricemia as compared with those without hyperuricemia (Table 5; all *p* < 0.05)

**Table 3:** Regression coefficients (B), 95% confidence intervals (CI), and *p*-values of multivariate regression analyses of hyperuricemia, hypertension, age, body mass index (BMI), serum creatinine, use of calcium channel blockers (CCB) and statins for platelet surface expression of activated glycoprotein (GP) IIb/IIIa in response to adenosine diphosphate (ADP), soluble CD40 ligand (sCD40L), light transmission aggregometry (LTA) in response to ADP, the VerifyNow P2Y12 assay, multiple electrode platelet aggregometry (MEA) in response to ADP, and the vasodilator-stimulated phosphoprotein (VASP) phosphorylation assay in clopidogrel-treated patients (*n* = 301)

	**Activated GPIIb/IIIa (MFI)**			**sCD40L (pg/mL)**			**LTA (maximal aggregation %)**		
	**B**	**CI**	***p***	**B**	**CI**	***p***	**B**	**CI**	***p***
Hyperuricemia	3.9	1.6–6.2	0.001	218.4	78.6–358.2	0.002	5.5	0.4–10.5	0.04
Hypertension	0.2	− 3.1–3.6	0.89	50.4	− 173.8–274.5	0.66	− 1.1	− 8.5–6.2	0.76
Age	0.1	0.05–0.2	0.003	0.2	− 5.6–5.9	0.96	0.3	0.1–0.5	0.001
BMI	0.3	0–0.5	0.05	− 15.6	− 30.8 to − 0.4	0.04	0.5	− 0.03–1.1	0.06
Creatinine	0.4	− 3.4–4.2	0.84	− 54.9	− 269–159.3	0.61	− 3.5	− 11.7–4.6	0.4
CCB	1.8	− 0.4–4	0.1	77.7	− 45.5–201	0.22	5.5	0.7–10.2	0.02
Statins	− 0.2	− 4.7–4.3	0.94	169.6	− 78.3–417.4	0.18	− 5	− 14.6–4.6	0.31
	**VerifyNow P2Y12 assay (PRU)**			**MEA (AU)**			**VASP assay (PRI)**		
	**B**	**CI**	***p***	**B**	**CI**	***p***	**B**	**CI**	***p***
Hyperuricemia	22.3	− 0.6–45.2	0.06	5.9	0.2–11.5	0.04	7.2	1.1–13.3	0.02
Hypertension	− 24.1	− 57.3–9.2	0.16	− 1.5	− 9.7–6.7	0.72	− 12.5	− 21.9 to − 3	0.01
Age	3	2.2–3.9	< 0.001	− 0.2	− 0.4–0.05	0.12	0.01	− 0.2–0.3	0.91
BMI	2.7	0.2–5.2	0.03	0.5	− 0.1–1.1	0.1	1.3	0.7–2	< 0.001
Creatinine	14.8	− 22.1–51.6	0.43	− 10.2	− 19.4 to − 0.9	0.03	3.8	− 5.8–13.5	0.44
CCB	18.7	− 2.8–40.2	0.09	2.6	− 2.8–7.9	0.35	4.9	− 0.8–10.6	0.09
Statins	11.8	− 31.7–55.3	0.59	− 0.1	− 11.1–11	0.99	4.7	− 6.9–16.3	0.42

*AU* aggregation units, *MFI* mean fluorescence intensity, *PRI* platelet reactivity index, *PRU* P2Y12 reaction units

**Fig. 2:**
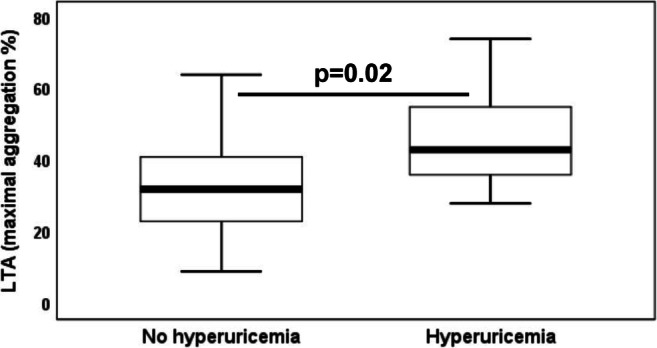
On-treatment residual adenosine diphosphate–inducible platelet reactivity by light transmission aggregometry in prasugrel-treated patients without and with hyperuricemia. The boundaries of the box show the lower and upper quartile of data, and the line inside the box represents the median. Whiskers are drawn from the edge of the box to the highest and lowest values that are outside the box but within 1.5 times the box length

In prasugrel-treated patients of group 2 (*n* = 118), on-treatment residual ADP-inducible platelet reactivity by LTA was significantly higher in those with hyperuricemia as compared with patients without hyperuricemia (Fig. 2; 43% (36–55%) vs. 32% (23–41%), *p* = 0.02), whereas levels of platelet aggregation by MEA were similar in patients without and with hyperuricemia (20 AU (15–23 AU) vs. 20 AU (12–22 AU), *p* = 0.7). The association between ADP-inducible platelet reactivity by LTA and hyperuricemia remained significant after adjustment for differences in patient characteristics by multivariate regression analysis (Table 4; *p* = 0.02). Moreover, there was a trend towards higher on-treatment residual ADP-inducible platelet reactivity by MEA in patients with hyperuricemia following multivariate regression analysis (Table 4; *p* = 0.06). HRPR by LTA and MEA was seen in 5 and 2 prasugrel-treated patients, respectively, and HRPR by LTA occurred more often in patients with hyperuricemia than in those without hyperuricemia (Table 6; *p* = 0.03).

**Table 4:** Regression coefficients (B), 95% confidence intervals (CI), and *p*-values of multivariate regression analyses of hyperuricemia, serum creatinine, platelet count and the use of calcium channel blockers (CCB) for light transmission aggregometry (LTA), and multiple electrode platelet aggregometry (MEA) in response to adenosine diphosphate in prasugrel-treated patients (*n* = 118)

	LTA (maximal aggregation %)			MEA (AU)		
	B	CI	p	B	CI	p
Hyperuricemia	11.6	1.7–21.5	0.02	4.2	− 0.2–8.6	0.06
Creatinine	1.6	− 5.7–8.9	0.66	− 4	− 7.3 to − 0.8	0.02
Platelet count	− 0.02	− 0.07–0.03	0.47	0.02	0–0.05	0.05
CCB	− 0.3	− 11.6–10.9	0.95	0.4	− 4.6–5.4	0.88

*AU* aggregation units

**Table 5:** High on-treatment residual platelet reactivity (HRPR) by light transmission aggregometry (LTA), the VerifyNow P2Y12 assay, multiple electrode platelet aggregometry (MEA), and the vasodilator-stimulated phosphoprotein (VASP) phosphorylation assay in clopidogrel-treated patients (n=301) without and with hyperuricemia. Measurements by LTA, the VerifyNow P2Y12 assay, MEA, and the VASP assay were available for 299, 300, 293, and 299 patients, respectively

	No hyperuricemia (*n* = 218)	Hyperuricemia (*n* = 83)	*p*
HRPR LTA	8.8%	18.3%	0.02
HRPR VerifyNow P2Y12 assay	39.6%	59%	0.003
HRPR MEA	33.6%	46.3%	0.04
HRPR VASP assay	41.2%	57.8%	0.01

**Table 6:** High on-treatment residual platelet reactivity (HRPR) by light transmission aggregometry (LTA) and multiple electrode platelet aggregometry (MEA) in prasugrel (*n* = 118)- and ticagrelor (*n* = 88)-treated patients without and with hyperuricemia. Measurements by LTA and MEA were available for all patients

	Prasugrel		*p*	Ticagrelor		*p*
	No hyperuricemia (*n* = 98)	Hyperuricemia (*n* = 20)		No hyperuricemia (*n* = 66)	Hyperuricemia (*n* = 22)	
HRPR LTA	2%	15%	0.03	3%	4.5%	1
HRPR MEA	1%	5%	0.31	0%	0%	1

In ticagrelor-treated patients of group 2, on-treatment residual ADP-inducible platelet reactivity by LTA and MEA did not differ significantly between patients without and with hyperuricemia in univariate analyses (LTA: 36% (28.5–42%) vs. 37% (24–45%), *p* = 0.91; MEA: 20 AU (15–25 AU) vs. 19 AU (14–22 AU), *p* = 0.15), and after adjustment for differences in patient characteristics by multivariate regression analysis (both *p* > 0.4). HRPR by LTA and MEA was observed in 3 and 0 ticagrelor-treated patients, respectively, and occurred to a similar extent in patients without and with hyperuricemia (Table 6; both *p* = 1).

## Discussion

Our study is the first to investigate the association of hyperuricemia with on-treatment platelet reactivity as assessed by flow cytometry and a variety of platelet aggregation tests in patients undergoing elective and acute angioplasty with stent implantation, respectively. Hyperuricemia was linked to decreased platelet inhibition by clopidogrel and prasugrel. Moreover, HRPR was seen more frequently in hyperuricemic patients receiving thienopyridines as P2Y12 inhibitors. In contrast, the antiplatelet effect of ticagrelor was similar in patients without and with hyperuricemia.

In line with the PLATO trial [16], we observed a strong trend towards higher levels of uric acid and a greater incidence of hyperuricemia in ACS patients receiving ticagrelor as compared with prasugrel-treated patients.

Platelet reactivity was assessed 1 day after elective angioplasty and stenting (group 1) and 3 days after acute PCI (group 2). This approach was chosen to (1) be able to measure platelet reactivity during the hospital stay in all patients and (2) to limit a potential influence of ACS on residual platelet reactivity.

On-treatment residual ADP-inducible platelet reactivity was assessed by different methods because previous studies revealed a poor agreement of different platelet function tests regarding the classification of patients as clopidogrel responders or non-responders [10, 17]. Activated GPIIb/IIIa is expressed on the platelet surface upon platelet activation, and as fibrinogen receptor subsequently facilitates the interaction of platelets with each other and with other blood cells [18]. It can therefore be considered as sensitive parameter of platelet activation at the time of blood sampling. sCD40L was assessed to also study the association of hyperuricemia with a soluble marker of platelet activation. The advantage of measuring platelet biomarkers in plasma instead of determining their expression on the platelet surface is their stability in plasma over time. In contrast, the surface expression of platelet activation markers represents only a snapshot at the time of blood sampling. In a previous study, we have shown that sCD40L correlates strongly with other platelet-derived soluble biomarkers like platelet factor-4 and thrombospondin-1 [19]. Accordingly, plasma levels of sCD40L may allow to capture continuously ongoing platelet activation [18]. While activated GPIIb/IIIa has been linked to the occurrence of ischemic outcomes following peripheral endovascular interventions [11], elevated sCD40L may predict future myocardial infarction and death in ACS patients [20, 21]. LTA represents the historical gold standard of platelet function testing and has repeatedly been associated with atherothrombotic events after PCI [14, 22]. The VerifyNow P2Y12 assay is a fast and standardized point-of-care test based on the principle of optical aggregometry but working with whole blood samples, thus avoiding time-consuming and error-prone centrifugation steps [23]. MEA is an adaptation of impedance aggregometry and a whole-blood near-point-of care test [24]. The VASP assay captures the inhibition of VASP phosphorylation by ADP, which is mediated by P2Y12 through the inhibition of adenylyl cyclase. Consequently, it represents a highly-specific method for assessing the effects of P2Y12 inhibitors [25]. Similar to LTA, the results of the VerifyNowP2Y12 assay, MEA, and the VASP assay have been shown to correlate with clinical outcomes following PCI [14].

The cut-off values for HRPR were based on the consensus document on the definition of high on-treatment platelet reactivity to ADP [14], and thereby on previous studies revealing an association of HRPR with ischemic events post PCI. However, it should be kept in mind that these thresholds were obtained in patients on clopidogrel therapy and may not be fully applicable to prasugrel- and ticagrelor-treated patients.

While on-treatment platelet reactivity to ADP was significantly higher in hyperuricemic patients receiving clopidogrel or prasugrel, no association between hyperuricemia and the response to ticagrelor was discernible. This observation suggests that the hepatic conversion of thienopyridines to their active metabolites may be impaired in hyperuricemia resulting in less pronounced P2Y12 inhibition [26, 27]. In contrast, ticagrelor as direct-acting P2Y12 antagonist exerts its antiplatelet effects independently of hepatic biotransformation [27]. Accordingly, the magnitude of ticagrelor-mediated platelet inhibition would not be impaired by effects of hyperuricemia on hepatic metabolism. In order to prove this hypothesis, it would be necessary to measure the active metabolites of clopidogrel and prasugrel in patients without and with hyperuricemia. Besides its direct mode of action, ticagrelor differs from clopidogrel and prasugrel regarding the binding site at the P2Y12 receptor [28]. Further, ticagrelor also inhibits the type 1 equilibrative nucleoside transporter (ENT1) [29], leading to an increase in extracellular adenosine concentrations with potential additive antiplatelet effects [30, 31]. Alternatively, higher levels of uric acid in ticagrelor-treated patients may be drug-related [16], which could explain the lack of an association between hyperuricemia and HRPR in patients receiving ticagrelor. Another explanation for higher on-treatment residual platelet reactivity in patients with elevated uric acid levels may be an overall increased inflammatory status in hyperuricemia [32, 33], possibly resulting in general platelet hyperreactivity [34]. However, patients without and with hyperuricemia in both groups of our study population had similar levels of hsCRP and WBC. Moreover, in case of pre-existing platelet hyperreactivity due to inflammation, increased on-treatment platelet reactivity to ADP should have also been present in ticagrelor-treated patients with hyperuricemia.

Due to the non-interventional design of our study, we cannot draw conclusions about therapeutic implications of our findings. However, our observations raise the question whether or not lowering uric acid increases the response to thienopyridines and, if yes, leads to better clinical outcomes in hyperuricemic patients with HRPR. Thus, clinical trials addressing the effect of uric acid lowering therapy on platelet reactivity and future ischemic events in patients with HRPR would be of great interest. Moreover, based on our findings one may speculate that hyperuricemic patients may particularly benefit from antiplatelet therapy with ticagrelor instead of prasugrel or clopidogrel. In this regard, it would be interesting to perform a subanalysis of the PLATO trial to investigate if there is a greater reduction of ischemic outcomes with ticagrelor vs. clopidogrel in hyperuricemic patients as compared with patients without hyperuricemia [16].

A limitation of our study is the lack of clinical outcome data. By postponing platelet function testing to day 3 after acute PCI, we sought to avoid an impact of ACS on residual platelet reactivity. However, we cannot completely rule out that platelet reactivity measurements in ACS patients were still influenced by the acute event. Furthermore, we cannot provide pre-procedural values or data on the variability of platelet reactivity over time. Levels of uric acid prior to the initiation of P2Y12 inhibitor therapy are not available in the study participants because all patients underwent angioplasty and received the respective P2Y12 inhibitor before blood sampling. Finally, we did not measure the active metabolites of clopidogrel and prasugrel and therefore cannot prove that hyperuricemia impairs the hepatic metabolism of thienopyridines.

In conclusion, hyperuricemia is associated with decreased platelet inhibition by thienopyridines but a normal response to ticagrelor. It remains to be established if lowering uric acid increases the antiplatelet effects of clopidogrel and prasugrel in hyperuricemic patients with HRPR. Moreover, future trials are warranted to investigate underlying mechanisms of our findings.

## Data Availability

All participants were enrolled at the Department of Internal Medicine II of the Medical University of Vienna.
